# Experiencing Nature through Immersive Virtual Environments: Environmental Perceptions, Physical Engagement, and Affective Responses during a Simulated Nature Walk

**DOI:** 10.3389/fpsyg.2017.02321

**Published:** 2018-01-23

**Authors:** Giovanna Calogiuri, Sigbjørn Litleskare, Kaia A. Fagerheim, Tore L. Rydgren, Elena Brambilla, Miranda Thurston

**Affiliations:** ^1^Department of Public Health, Faculty of Social and Health Sciences, Inland Norway University of Applied Sciences, Elverum, Norway; ^2^Inland Norway School of Sport Sciences, Faculty of Social and Health Sciences, Inland Norway University of Applied Sciences, Elverum, Norway; ^3^IT Department, Inland Norway University of Applied Sciences, Elverum, Norway; ^4^Department of Biomedical Science for Health, University of Milan, Milan, Italy

**Keywords:** environmental perception, green exercise, physical activity promotion, restorative environments, virtual reality

## Abstract

By combining physical activity and exposure to nature, *green exercise* can provide additional health benefits compared to physical activity alone. Immersive Virtual Environments (IVE) have emerged as a potentially valuable supplement to environmental and behavioral research, and might also provide new approaches to green exercise promotion. However, it is unknown to what extent green exercise in IVE can provide psychophysiological responses similar to those experienced in real natural environments. In this study, 26 healthy adults underwent three experimental conditions: nature walk, sitting-IVE, and treadmill-IVE. The nature walk took place on a paved trail along a large river. In the IVE conditions, the participants wore a head-mounted display with headphones reproducing a 360° video and audio of the nature walk, either sitting on a chair or walking on a manually driven treadmill. Measurements included environmental perceptions (presence and perceived environmental restorativeness – PER), physical engagement (walking speed, heart rate, and perceived exertion), and affective responses (enjoyment and affect). Additionally, qualitative information was collected through open-ended questions. The participants rated the IVEs with satisfactory levels of ‘being there’ and ‘sense of reality,’ but also reported discomforts such as ‘flatness,’ ‘movement lag’ and ‘cyber sickness.’ With equivalent heart rate and walking speed, participants reported higher perceived exertion in the IVEs than in the nature walk. The nature walk was associated with high enjoyment and enhanced affect. However, despite equivalent ratings of PER in the nature walk and in the IVEs, the latter were perceived as less enjoyable and gave rise to a poorer affect. Presence and PER did not differ between the two IVEs, although in the treadmill-IVE the negative affective responses had slightly smaller magnitude than in the sitting-IVE. In both the IVEs, the negative affective responses were mainly associated with cyber sickness, whereas PER was positively associated with enjoyment. From the qualitative analysis, it emerged that poor postural control and lack of a holistic sensory experience can also hinder immersion in the IVE. The results indicate that IVE technology might in future be a useful instrument in green exercise research and promotion, but only if image quality and cyber sickness can be addressed.

## Introduction

By combining physical activity and exposure to nature, green exercise can provide several health benefits (Pretty et al., 2003). Studies have, for example, shown that green exercise can provide greater benefits compared to physical activity performed indoors or in an urban setting, which include a reduction in psychophysiological stress and enhanced mental health (Bowler et al., 2010; Thompson Coon et al., 2011). In particular, a meta-analysis (Bowler et al., 2010) showed that green exercise studies consistently found significant reductions in negative emotional states such as fatigue, anger and sadness. Green exercise has also been consistently associated with lower perceived exertion compared to exercising indoors while at the same time inducing people to engage in more vigorous physical activity (Focht, 2009; Calogiuri et al., 2015). This implies that green exercise can increase the likelihood of higher exercise intensities being reached, which in turn can lead to a number of health benefits (Gladwell et al., 2013).

The attention-restoration theory (ART) of Kaplan (1989, 1995) has been used to explain the positive psychological effects of green exercise. ART postulates that some environments can elicit restoration from mental fatigue by triggering a spontaneous (and therefore effortless) form of attention, which is referred to as *fascination*. Some specific features of the natural world such as clouds in the sky or leaves in a breeze are hypothesized to have particular advantages in prompting attention-restoration mechanisms. Moreover, being outdoors in a natural environment can provide a sense of *being away* from everyday problems, thus contributing to restorative experiences. The theory specifies two additional components: *extent* and *compatibility*, the former representing the degree to which an environment is perceived as being coherently ordered and having substantial scope, while the latter represents the degree to which the environment matches a person’s inclinations at the time. A number of studies have found that exercising in natural environments has greater potential for restoration compared to indoor (Hug et al., 2009; Calogiuri et al., 2016a) and urban (Bodin and Hartig, 2003; Hartig et al., 2003) environments, while also giving rise to improved cognitive performance (Hartig et al., 1991, 2003), enhanced psychological states (Hartig et al., 1991, 2003; Calogiuri et al., 2015), and reduction of psychophysical stress (Hartig et al., 2003; Aspinall et al., 2015; Calogiuri et al., 2015).

Immersive Virtual Environments (IVEs) consist of synthetic sensory information that provide a surrounding and continuous stream of stimuli, creating the illusory perception of being enclosed within and interacting with a real environment (Loomis et al., 1999; Smith, 2015). IVEs are becoming increasingly popular, especially in the form of head-mounted displays (HMD), a device with a motion sensor that allows a 360° vision of a virtual world while eliminating the visual contact with external reality. The popularity of IVEs and HMDs follows the introduction of relatively affordable technology that not only provides the opportunity to immerse oneself in pre-set IVEs, but also allows the creation of new IVEs using special 360° cameras and freely available and customizable applications. One of the potential advantages of HMD is that they can provide relatively intense immersive experiences. In IVE sciences, immersion is defined as the extent to which a computer-generated environment is “capable of delivering an inclusive, extensive, surrounding, and vivid illusion of reality to the senses of a human participant” (Slater and Wilbur, 1997), and it is commonly evaluated by assessing participants’ feelings of presence. The concept of presence, i.e., the subjective feeling of “being in the virtual environment” (Slater and Wilbur, 1997), is therefore a key element in research related to the effectiveness of virtual reality technology, including (but not limited to) its application in the physical activity and exercise sciences (Pasco, 2013).

Green exercise research faces a number of challenges, especially in relation to the extent to which studies can control for possible confounders when comparing indoor and outdoor environments (Lee and Maheswaran, 2011; Rogerson et al., 2016). Different weather conditions and terrains (e.g., a paved trail as opposed to a treadmill), for example, might lead to differences in physical engagement and influence psychophysiological responses. IVEs, however, can engage research participants in highly controlled immersive environmental experiences (Smith, 2015). Furthermore, IVE could, in the future, provide a simple way of integrating experiences of nature into people’s everyday lives, as well as supplement rehabilitation and health promotion programs: in an urbanized society, a large number of individuals do not (or cannot) engage in green exercise on a regular basis: recent estimates show that in Norway, for instance, almost half of the population do not engage in any green exercise in a typical week (Calogiuri et al., 2016b), while in the United Kingdom this reaches 80% (White et al., 2016). Yet the application of and research into this technology in relation to environmental or exercise sciences is still in its infancy. In particular it is not clear, in terms of participants’ perceptions, to what extent IVE technology can reproduce life-like experiences of green exercise. Research suggests, for example, that watching images or videos of nature can provide a similar, although smaller, burst of positive affect compared with a walk in real nature (Plante et al., 2006; Mayer et al., 2009). Furthermore, positive psychophysiological and cognitive effects have also been demonstrated in a study by Valtchanov et al. (2010), in which the participants were exposed to a virtual environment constructed as a photo-realistic forest (i.e., a high quality computer-generated representation of a forest). However, to the best of our knowledge, no research has yet investigated how people respond and interact with IVEs that are more encompassing and dynamic, such as watching a first-person 360° video of a nature walk.

Engaging in physical activity while being exposed to virtual nature might provide additional benefits: physical movement might contribute to more positive affective responses as compared with a sedentary exposure to virtual nature, as in fact physical activity alone is known to provide affective benefits (Ekkekakis et al., 2011); having the possibility of moving might also elicit more immersive experiences in the IVE, as this might provide greater engagement with the virtual environment; furthermore, physical movement might prevent discomfort caused by the gap between the movements of virtual self and the movements of the real self. Studies have previously tested experimental conditions in which participants exercised on a treadmill or a stationary bike while watching images or videos of nature displayed on a screen (Pretty et al., 2005; Plante et al., 2006; Akers et al., 2012; White et al., 2015; Yeh et al., 2017). However, despite attempts within the gaming industry to combine HMDs with special ergometers and other devices, how best to combine IVE and physical movement in a controlled research environment remains underexplored. Since the 1990s, using different types of IVE technology, researchers have studied how to integrate physical movement with exposure to IVEs and how IVEs can influence people’s physical activity patterns (Slater et al., 1995; Jaffe et al., 2004; Sheik-Nainar and Kaber, 2007; Peruzzi et al., 2016). However, to the best of our knowledge, few of these studies have attempted to combine physical activity with HMDs and none of them has investigated whether the additional component of physical movement can actually elicit feelings of presence or positive psychological states to a greater extent than a sedentary exposure. Besides the interest in understanding the extent to which physical movement can elicit more immersive experiences, it is also important to consider the effects that exercising in IVE conditions might have on the way people move and exercise. Wearing a HDM might, for example, lead participants to walking or exercising at a slower pace than they would normally do in a real natural environment, reducing some of the potential benefits of simulated green exercise experiences. Moreover, because the subjective experience of exercise intensity is often associated with health outcomes as well as motivation for regular exercise (Ekkekakis et al., 2011), it is important to consider people’s responses to simulated green exercise in terms of perceived exertion.

The purpose of the current study was to investigate the extent to which commercially available IVE technology used under laboratory conditions can simulate green exercise experience, reproducing similar psychophysiological responses. In addition, we investigated whether physical movement (i.e., walking on a treadmill) could elicit greater engagement with the virtual natural environment, leading to higher positive affective responses compared to sedentary exposure.

## Materials and Methods

### Participants

Participants were recruited among students and employees at the Faculty of Social and Health Sciences at the Inland Norway University of Applied Sciences through announcements on the University’s webpage and presentations to students during classes. The inclusion criteria for participation were: (1) age 20–45 years; (2) able to walk for 10 min outdoors and on a treadmill; (3) not being an elite athlete (i.e., individuals currently competing in sports at a national level or above). Initially, 65 individuals responded to the researchers’ invitation, 34 of whom met the inclusion criteria and confirmed their intention to participate in the study. Eight individuals dropped-out (i.e., did not attend on the scheduled day of the experiment). Thus, the final sample included 26 participants (14 males, 12 females; age: 26 ± 8 years; BMI: 23.12 ± 5.03), all of whom completed the full set of experiments and assessments. All participants were informed in writing about the purpose of the study and associated risks before they provided their written consent. The study was approved by the Norwegian Centre for Research Data and was performed according to the Declaration of Helsinki.

### IVE Technology

The IVE was constructed as a 360° video reproducing a nature walk in the exact same location used for the ‘outdoor walk’ condition; this allowed us to reduce confounders such as different characteristics of the two environments (see section “Design and Procedure”). The video was filmed using a Samsung gear 360 sm-c200 camera 2 days before the beginning of the experimentations. The audio was recorded simultaneously in order to capture sounds such as footsteps, the voices of people passing by and other natural events. The camera was mounted on a modified Yelangu s60t handheld stabilizer. The video was then run through two software stabilizing programs – first in Adobe After Effects CC 2017, Warp Stabilizer VFX and then in Samsung Gear 360 ActionDirector, build 1.0.0.2423, in order to further improve the stability of the images in the post-production phase. Finally, the video was adjusted for being viewed using a 360 VR video in Samsung Gear 360 ActionDirector. The playback was made via Samsung S7, with Android 7.0, mounted on a Samsung Gear VR mask. To reproduce the sounds and minimize external noises, during the experimentation participants wore a Sennheiser HD 201 headset together with the head-mounted display.

### Design and Procedure

**FIGURE 1:**
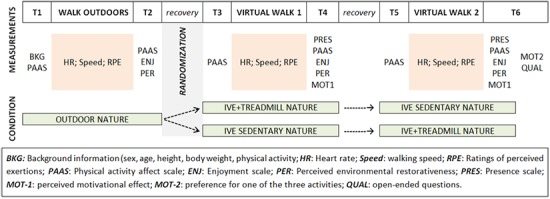
Experimental design and data gathering organization.

**FIGURE 2:**
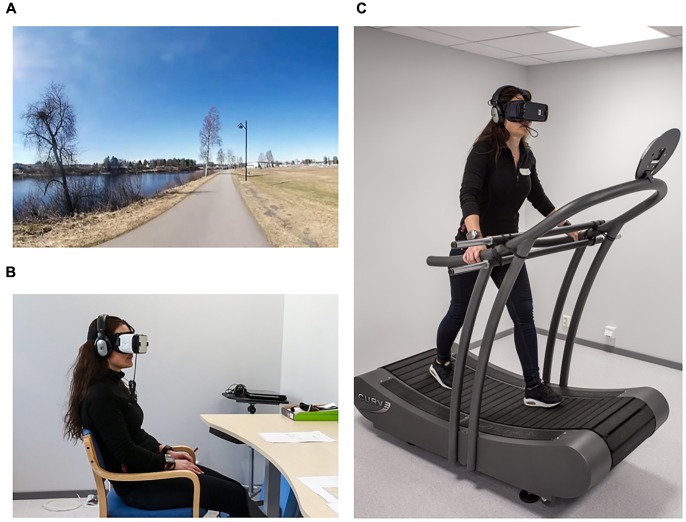
Experimental conditions: **(A)** Outdoor (walk in real nature); **(B)** Sitting-IVE (sedentary exposure to virtual walk in nature); **(C)** Treadmill-IVE (virtual walk in nature while walking on a manually activated treadmill). Written informed consent was obtained from the individual for the publication of these images.

A schematic overview of the experimental design and data collection process is shown in **Figure 1**. All participants underwent three conditions: (a) a walk outdoors in a natural environment, (b) a sedentary exposure to a IVE video, and (c) a treadmill walk whilst being exposed to the same IVE video (**Figure 2**). Each condition lasted 10 min, as this span was previously shown to provide the largest effects on psychological outcomes in green exercise experiments (Barton and Pretty, 2010). Furthermore, according to the World Health Organization’s guidelines, bouts of at least 10 min constitute the minimum unit for health-enhancing physical activity (WHO, 2017). All participants completed the walk outdoors before undergoing the two other conditions, which were administered in a randomized counter-balanced order. Each participant underwent all three conditions on the same day, with a minimum break of 15 min provided between each condition in order for participants to recover from potential discomfort. After such time, participants were asked whether they felt sufficiently recovered and were comfortable to proceed with the experimentation, and additional resting time was provided if required. All experiments took place in the period between May 2nd and 10th 2017, with the IVE video recorded 2 days before the first session. The outdoor weather condition varied from sunny to overcast, with the temperature ranging between 7 and 17°C. The weather during the filming was sunny with a gentle breeze, which could be heard at times in the playback. The temperature in the laboratory was kept constant at 21°C.

The outdoor walk took place on a fairly straight paved trail along a large river in proximity to the university, where the IVE conditions were administered in the laboratory. The environment also included some built elements, such as buildings and a football field. The participants met the researchers in a building by the trail and were individually accompanied by one of the researchers to the starting point of the walk. The participants were equipped with a wristwatch with a heart rate monitor and GPS (Garmin, Forerunner 310XT), which had an alarm set-up for ringing after 5 min. They were instructed to walk at a comfortable pace on the trail until the alarm rang, at which point they turned around and walked back to the starting point. At completion of the outdoor walk, the participants were accompanied to the laboratory. In the treadmill condition, the participants walked on a manually driven treadmill (Woodway, Curve) equipped with a structure for the participants to hold on to by placing their hands in front of them. Unlike engine-driven treadmills, manually driven treadmills are activated by a person moving their feet while walking, similar to what happens when walking over ground. In this way, the participants could control their pace in a spontaneous manner. All participants underwent a short trial of walking on the treadmill before starting the IVE condition. In the sitting condition, the participants sat on a chair, in a separate room within the laboratory.

### Instruments

#### Environmental Perceptions

Perceived environmental restorativeness was measured after completion of each condition using two subscales of the Perceived Restorativeness Scale (Hartig et al., 1997): ‘fascination’ (five items) and ‘being away’ (two items). The components ‘extent’ and ‘compatibility’ were not used, as preliminary testing suggested that these two items were not applicable to the IVE conditions and might have led to inaccurate assessments. Each item was rated on an 11-point Likert scale (0 = absolutely disagree, 10 = absolutely agree). When these questions were administered after the IVE conditions, a caption explicitly indicated “The following questions relate to the virtual environment.” The scale showed, in general, adequate internal consistency for ‘fascination’ (α = 0.85–0.92), though poorer internal consistency was detected for the component ‘being away’ (α = 0.56–0.87). Additionally, eight items were used to assess the participants’ feeling of presence after the two IVE conditions. Seven of these items were adapted from those used by Nichols et al. (2000), while an additional item was included that related to the extent to which participants experienced cyber sickness (**Table 1**). The items were formulated as statements, each participant being asked to rate the extent to which they agreed with each of them on an 11-point Likert scale (0 = absolutely disagree, 10 = absolutely agree).

**Table 1:** Items used to assess presence in participants who underwent an IVE-based ‘nature walk’^a^.

Short name	Item
Being there	In the computer generated world I had the sense of ‘being there’
Realism	I thought of the virtual environment as equal to the real environment
Sense of reality	The virtual world became more real or present to me compared to the real world. NB: by ‘real world’ we mean the room where you were undergoing the test
Awareness	During the ‘virtual walk,’ I often thought of the other person(s) in the room with me
Other persons	It would have been more enjoyable to engage with the ‘virtual world’ with no-one else in the room
External noises	Whilst I was doing the ‘virtual walk,’ I paid much attention to other noises around me in the room
Flatness	The virtual world appeared flat and missing in depth
Movement lag	The lag or delay between my movements and the moving in the ‘virtual walk’ were disturbing
Cyber sickness	During the ‘virtual walk’ I got dizzy


*^a^Each participant was exposed to the same IVE-based ‘nature walks,’ once while they sat on a chair and once while they walked on a manual treadmill.*

#### Physical Engagement

Heart rate (HR) was continuously measured during all experimental conditions using a HR-monitor (Garmin, Forerunner 310XT), while ratings of perceived exertion (RPE) were measured immediately after completing each experimental condition using a Borg scale in a 20-point version (Borg, 1982). The walking speed was also recorded using the Garmin GPS and the treadmill computer in the outdoor and treadmill conditions, respectively.

#### Affective Responses

Enjoyment was measured after each experimental condition using a single item question: “On a scale from 0 to 10, how enjoyable is the activity you have engaged in?” Participants gave their answer on a numbered line (0 = not enjoyable at all; 10 = absolutely enjoyable). Additionally, participants’ affective responses were assessed by administering the Physical Activity Affect Scale (PAAS) (Lox et al., 2000) immediately before and immediately after undergoing each experimental condition. The PAAS consists of 12 items corresponding to different emotions (e.g., “energetic,” “calm,” “miserable,” and “tired”) and placed them within four quadrants, in line with Russell’s circumplex model of affect and arousal (Russell, 1980): positive affect, tranquility, negative affect, and fatigue. Each item was measured on a 5-point rating scale (0 = strongly disagree; 4 = strongly agree). Reliability analysis, showed reasonably adequate internal consistency for most assessments (α = 0.64–0.86), though somewhat poor levels of internal consistency were detected for negative affect in the pre-condition assessments (α = 0.46–0.52).

#### Qualitative Data

As little is known about how people respond to virtual experiences of nature, especially in relation to the technology used in this particular study, qualitative information was collected using a series of open-ended questions, which were presented to the participants after completion of all three conditions and quantitative measurements. Such questions, to which the participants responded in written form, were inspired by the structure of the quantitative assessments: a question was developed for each of the quantitative variables in order to explore the meaning behind participants’ responses in more detail, for example: “*In the questionnaire, you were asked to report the extent to which you felt the environments were ‘fascinating’ and gave you feelings of ‘being away.’ Could you say how well (or how poorly) did the IVE video reproduce such characteristics, compared with the outdoor/real environment?*” and “*When you answered the question about how ‘enjoyable’ the activity was, what determined where in the scale you put your mark? Please, describe the feelings you experienced in all three conditions separately*.”

### Analyses

Data were first explored for distribution, possible outliers and missing values. A one-way repeated measurements analysis of variance (ANOVA) was used to establish possible effects of ‘condition’ (i.e., outdoor, sitting, and treadmill) for the different study variables. For the PAAS components, a factorial (two-way) repeated ANOVA was used to investigate possible pre-post changes in interaction with the experimental conditions. If significance was achieved in the within-subjects test, a *post hoc* analysis with Bonferroni’s adjustment of alpha was applied in order to examine possible differences across the individual conditions. Additionally, Spearman’s rank correlation coefficient (ρ) was used to examine possible associations among all study variables. The PAAS components were run into the correlation analysis in form of *delta* values (i.e., the difference between post-values and pre-values). All statistical analyses was carried out using IMB Statistics SPSS version 21 (IBM Corp., New York). Significance was set at *p* < 0.05.

The qualitative data were analyzed in accordance with the ‘framework approach’ (Gale et al., 2013), which provides clear steps for summarizing qualitative data in a way that sheds light on the participants’ responses to the quantitative questions. The method is systematic and transparent and provides a clear trail from raw data to thematic codes and quotations. These aspects of the method contribute toward evaluating the trustworthiness of the analysis. In addition, the process allows for the inclusion of more than one researcher at various points to discuss the emerging framework of codes, categories and themes. In this study, discussion took place between three members of the team in order to arrive at a more refined version of comments. Initially, a coding frame relating to the different overarching domains of the questionnaire was used (i.e., presence, perceived environmental restorativeness, physical engagement, and affective responses). Reiterative reading and recoding of the data led to refinement of the coding frame and the development of overarching themes.

## Results

### Presence and Perceived Environmental Restorativeness

**FIGURE 3:**
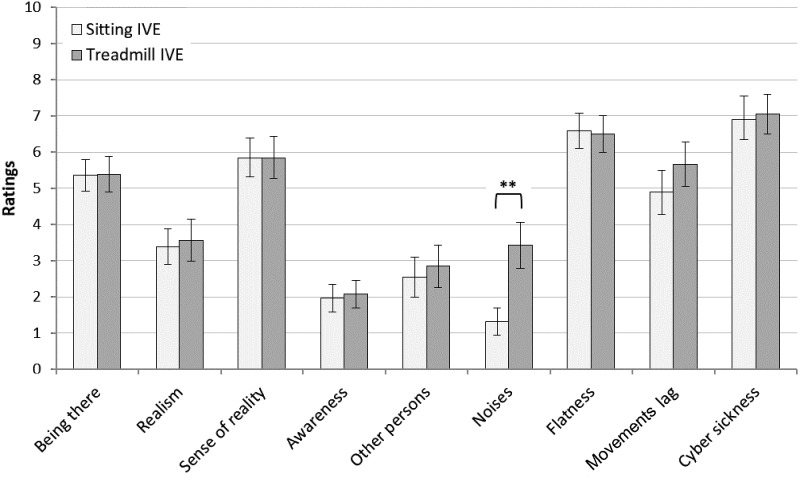
Ratings of *presence* in a ‘sitting-IVE’ condition and a ‘treadmill-IVE’ condition (M ± SE; *n* = 26, repeated measurements). ^∗∗^*p* < 0.001 in a *post hoc* comparison of sitting vs. treadmill.

No significant difference among the three conditions was found for the two components of perceived environmental restorativeness, ‘fascination’ [*F*(2,22) = 2,89; *p* = 0.076] and ‘being away’ [*F*(2,22) = 2.41; *p* = 0.112]. In relation to the feelings of presence assessed in concomitance with the IVE conditions, the participants reported high ratings of ‘flatness’ medium-high ratings of ‘being there’ and ‘sense of reality,’ low levels of realism as well as low levels for the items depicting external disturbances such as ‘awareness,’ ‘other persons,’ and ‘noises.’ Furthermore, the participants reported quite high ratings of ‘movement lag’ and especially ‘cyber sickness’ (**Figure 3**). The ANOVA showed no significant difference between the sitting and the treadmill condition for all the presence domains, apart from ‘noises’ [*F*(2,24) = 11.60; *p* = 0.002], which had significantly higher ratings in the treadmill condition compared with the sitting condition.

Significant correlations were found among the different domains of perceived environmental restorativeness and presence, though different patterns of association emerged in the sitting and the treadmill conditions (**Table 2**). ‘Fascination’ and ‘being away’ were highly correlated with each other in both the sitting and the treadmill conditions. ‘Fascination’ was positively associated with ‘being there’ and ‘realism’ in both the sitting and the treadmill conditions, whereas it was positively associated with ‘sense of reality’ and negatively associated with ‘awareness’ only in the treadmill condition. ‘Being away’ was positively associated with ‘realism’ in both, the sitting and the treadmill conditions, while it was associated with ‘being there’ only in the sitting condition and with ‘sense of reality’ and ‘other persons’ only in the treadmill condition. Moreover, in the sitting condition, ‘being there’ was positively correlated with ‘realism’ and ‘sense of reality,’ while ‘awareness’ was positively correlated with ‘noises.’ In the treadmill condition, ‘being there’ was negatively correlated with ‘awareness’ and ‘movement lag,’ ‘movement lag’ was positively correlated with ‘flatness’ and ‘cyber sickness,’ and ‘flatness’ and ‘cyber sickness’ were positively correlated with each other.

**Table 2:** Spearman’s correlation among different domains of environmental perceptions in participants exposed to an IVE video while sitting on a chair and while walking on a manually driven treadmill (M ± SE; *n* = 26, repeated measurements).

Sitting IVE	Fascination	Being away	Being there	Realism	Sense of reality	Awareness	Other persons	Noises	Flatness	Movement lag
Being away	0.76**									
Being there	0.70**	0.72**								
Realism	0.71**	0.44*	0.66**							
Sense of reality	0.19	0.19	0.43*	0.15						
Awareness	-0.09	0.04	-0.07	0.14	0.18					
Other persons	0.34	0.32	0.14	0.23	0.21	0.38				
Noises	-0.10	0.03	-0.21	0.11	0.04	0.73**	0.38			
Flatness	0.18	0.13	0.06	0.18	-0.17	0.25	0.14	0.15		
Movement lag	-0.19	0.02	-0.36	-0.30	0.04	0.18	0.32	0.22	0.16	
Cyber sickness	-0.20	-0.36	-0.06	-0.13	0.17	0.00	0.07	-0.32	0.20	0.11

**Treadmill IVE**	**Fascination**	**Being away**	**Being there**	**Realism**	**Sense of reality**	**Awareness**	**Other persons**	**Noises**	**Flatness**	**Movement lag**

Being away	0.73**									
Being there	0.54**	0.38								
Realism	0.51**	0.39*	0.37							
Sense of reality	0.41*	0.59**	0.37	0.31						
Awareness	-0.39*	-0.25	-0.39*	-0.27	0.07					
Other persons	0.14	0.41*	-0.11	0.18	0.35	0.28				
Noises	-0.04	0.05	-0.38	0.09	-0.13	0.38	0.27			
Flatness	0.02	0.02	-0.30	-0.04	-0.14	0.18	0.00	0.24		
Movement lag	-0.19	-0.22	-0.43*	-0.18	-0.01	0.17	0.26	-0.09	0.39*	
Cyber sickness	-0.07	-0.10	-0.29	0.00	-0.16	0.24	0.00	0.12	0.44*	0.47*


*^∗^Correlation is significant at the 0.05 level (2-tailed). ^∗∗^Correlation is significant at the 0.01 level (2-tailed).*

The qualitative data supported the quantitative results, showing that a number of factors could disrupt the sense of presence: the noise of the treadmill (*n* = 9; e.g., “*The noise from the treadmill was way too loud*”), the lag between the pace of the individual and the pace in the IVE video (*n* = 13; e.g., “*The discrepancy in the movements gave me a feeling of not having control*”), cyber sickness or other physical discomforts (*n* = 19; e.g., “*It made me dizzy and sick*”), and the poor quality of the imaging (*n* = 21; e.g., “*The video was very blurry*”). The poor quality of the video was especially related by several participants with other elements of presence, such as cyber sickness (*n* = 4; e.g., “*The poor quality of the video made me [feel] sick*”), a feeling of (not) ‘being there’ (*n* = 6; e.g., “*The poor quality of the video made it less real*”), and to a certain extent the perceived environmental restorativeness (*n* = 1; e.g., “*The [settings in the] IVE were fascinating, but the poor quality of the video reduced their potential*”). It also emerged that because the IVE conditions only provided visual and auditory cues, it tended to reduce the achievement of a comparative outdoor nature experience (*n* = 5; e.g., “*Air, smell, vision. [In the IVE conditions] I felt deprived of the elements of nature and senses*”). The additional element of movement (treadmill condition) did not appear to have helped people feel more engaged with the natural environment, although in some cases it elicited greater feelings of ‘being there’ (*n* = 2; e.g., “*[In the treadmill condition] you could really feel that you were in that place because you can move while you are watching the video*”). On the other hand, the element of movement did not seem to provide a consistent protection from experiencing cyber sickness; in fact, only four participants reported they felt less sick in the treadmill condition than in the sitting condition, while two reported the opposite, and the remaining reported that they felt sick in “both IVE conditions” (*n* = 13).

### Physical Engagement

**FIGURE 4:**
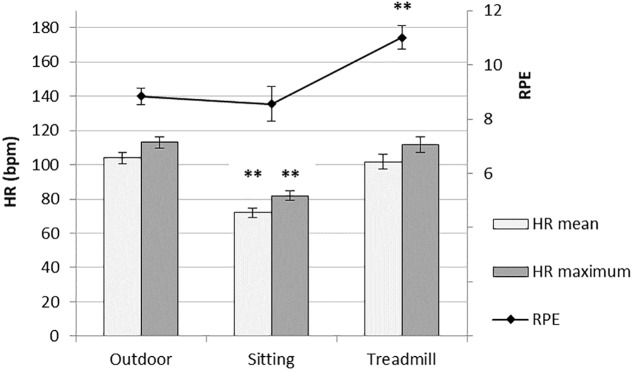
Heart rate (mean and maximum) and Ratings of perceived exertion (RPE) in a walk outdoors, a ‘sitting-IVE’ condition, and a ‘treadmill-IVE’ condition (M ± SE; *n* = 26, repeated measurements). ^∗∗^*p* < 0.001 in a *post hoc* comparison of the sitting or treadmill conditions with the outdoor condition, applying Bonferroni’s adjustment of alpha.

Significant differences across conditions for HR mean [*F*(2,24) = 70.84; *p* < 0.001] and HR max [*F*(2,24) = 71.71; *p* < 0.001] were found. The pairwise comparison found a significant difference when comparing the outdoor condition with the sitting condition (*p* < 0.001 for both variables), but not with the treadmill condition. Significant differences were also found when comparing the two IVE conditions with each other, with higher HR values in the treadmill condition as compared with the sitting (*p* < 0.001 for both variables; **Figure 4**). There were no differences in speed [min/km; *F*(1,25) = 3.52; *p* = 0.072] when comparing the outdoor and the treadmill condition. On the other hand, a significant effect across conditions was found for RPE [*F*(2,23) = 17.84; *p* < 0.001], with higher RPE values in the treadmill condition compared with both the outdoor (*p* < 0.001) and the sitting condition (*p* = 0.003), while no significant difference was found between the outdoor and the sitting condition (**Figure 4**). As shown in **Table 3**, both HR mean and HR max were positively associated with ‘movement lag’ in the sitting condition, while in the treadmill condition, RPE and HR mean were positively correlated with ‘cyber sickness.’

**Table 3:** Spearman’s correlation of the different domains of presence and perceived environmental restorativeness with affective responses and physical engagement in participants exposed to an IVE video while sitting on a chair and while walking on a manual treadmill (M ± SE; *n* = 26, repeated measurements).

Sitting IVE	Fascination	Being away	Being there	Realism	Sense of reality	Awareness	Other persons	Noises	Flatness	Movement lag	Cyber sickness
HR mean	0.20	0.31	-0.02	-0.24	-0.03	-0.02	0.23	-0.03	0.24	0.45*	0.13
HR max	0.16	0.29	-0.00	-0.23	0.03	0.02	0.21	-0.07	0.40*	0.50**	0.23
RPE	-0.29	-0.44*	-0.10	-0.37	0.13	-0.15	-0.01	-0.33	-0.38	-0.11	0.38
Enjoyment	0.47*	0.46**	0.38	0.36	-0.10	0.03	0.02	0.18	-0.08	-0.24	-0.79**
Pos. affect (delta)	-0.09	0.09	-0.17	-0.16	-0.31	0.03	-0.01	0.19	-0.24	-0.15	-0.73**
Tranquility (delta)	0.18	0.29	0.10	0.46*	-0.34	0.20	0.05	0.45*	0.21	-0.15	-0.60**
Neg. affect (delta)	0.00	-0.12	0.07	0.11	0.25	-0.05	0.08	-0.20	-0.02	0.08	0.77**
Fatigue (delta)	0.39*	0.33	0.34	0.44*	0.02	0.10	0.27	0.10	0.20	0.04	0.05

**Treadmill IVE**	**Fascination**	**Being away**	**Being there**	**Realism**	**Sense of reality**	**Awareness**	**Other persons**	**Noises**	**Flatness**	**Movement lag**	**Cyber sickness**

Speed	0.07	0.06	0.37	-0.22	0.08	-0.13	-0.05	-0.18	-0.20	-0.35	-0.33
HR mean	0.08	-0.07	-0.18	0.07	0.16	0.29	0.21	0.28	0.11	0.30	0.40*
HR max	0.07	-0.07	-0.24	0.06	0.09	0.29	0.23	0.36	0.11	0.28	0.36
RPE	-0.16	-0.10	-0.05	0.17	-0.01	0.32	0.06	0.15	-0.08	0.16	0.41*
Enjoyment	0.54**	0.40*	0.80**	0.26	0.42*	-0.17	0.04	-0.23	-0.34	-0.62**	-0.52**
Pos. affect (delta)	0.14	0.14	0.02	0.19	0.09	-0.55**	-0.06	0.02	-0.16	-0.26	-0.56**
Tranquility (delta)	0.03	0.10	-0.08	0.01	-0.12	-0.51**	-0.17	-0.13	-0.17	-0.16	-0.52**
Neg. affect (delta)	0.25	0.32	-0.12	0.18	0.21	0.22	0.30	0.13	0.15	0.24	0.65**
Fatigue (delta)	-0.02	-0.10	0.05	-0.29	-0.11	0.26	0.06	0.00	-0.03	0.12	0.43*


*^∗^Correlation is significant at the 0.05 level (2-tailed). ^∗∗^Correlation is significant at the 0.01 level (2-tailed). *HR* = Heart rate; *RPE* = Ratings of perceived exertion.*

From the qualitative data it emerged that the possibility of walking while being exposed to the IVE provided a ‘sense of liberation’ which made the participants feel less passive and more engaged with the virtual experience (*n* = 8; e.g., “*[In the treadmill condition] it was much better because I could move”; “[The sitting condition was] challenging and stressful as you can’t move*”). On the other hand, some participants reported physical discomforts due to poor postural control during the treadmill condition (*n* = 4; e.g., “*[The treadmill condition] was very stressful and tiring because I had to hold on to the handlebar very hard*”).

### Affective Responses

**Table 4** shows descriptive statistics for the affective responses, alongside the outcomes of the ANOVA and *post hoc* analysis. The ANOVA found significant differences across conditions for enjoyment, with a *post hoc* analysis showing that compared with the outdoor walk participants reported significantly less enjoyment in both the sitting and the treadmill conditions. The ANOVA also showed a significant interaction of ‘pre-post’ by ‘condition’ for positive affect, negative affect, and fatigue, whereas the interaction was not significant for tranquility. The affect profile assessed before the nature walk showed that the participants reported low ratings of negative affect, fatigue, and positive affect, whereas higher ratings were recorded for tranquility. A *post hoc* analysis applying a Bonferroni’s correction of alpha showed an improvement of the affect profile after completing the outdoor walk, with a significant reduction of the ratings of negative affect and fatigue. In contrast, the profile of affect worsened after both IVE conditions, with a slightly larger magnitude in the sitting condition: the ratings for positive affect and tranquility reduced (change significant in both conditions), whereas the ratings of negative affect and fatigue increased (change significant only in the sitting condition). A *post hoc* comparison on delta values across the different conditions showed a significant difference between the outdoor walk and both the IVE conditions for all PAAS components, whereas when comparing the two IVE conditions with each other, it was found that the reduction in positive affect was significantly larger in the sitting than in the treadmill condition.

**Table 4:** Affective responses to a walk outdoors in a real natural environment and two virtual nature walks (M ± SD; *n* = 26).

	Outdoor walk	Sitting IVE	Treadmill IVE	Pre vs. Post	Condition	Interaction
**Enjoyment**	7.69 ± 1.78	3.00 ± 2.59^a^	3.96 ± 2.32^a^	-	*F*(2,24) = 29.93^∗∗^	-
**Positive affect**	
*Pre*	0.68 ± 0.16	0.62 ± 0.25^b^	0.57 ± 0.24^b^	*F*(1,25) = 25.304^∗∗^	*F*(2,50) = 20.232^∗∗^	*F*(2,50) = 14.836^∗∗^
*Post*	0.70 ± 0.19	0.43 ± 0.25	0.49 ± 0.22			
*Delta*	0.02 ± 0.10	-0.19 ± 0.17^ac^	-0.08 ± 0.15^ac^			
**Tranquility**	
*Pre*	2.82 ± 0.91	2.59 ± 0.80^b^	2.54 ± 0.84^b^	*F*(1,25) = 20.346^∗∗^	*F*(2,50) = 14.114^∗∗^	*F*(2,50) = 6.550
*Post*	2.83 ± 0.75	2.03 ± 1.06	1.99 ± 0.89			
*Delta*	0.01 ± 0.67	-0.56 ± 0.69^a^	-0.55 ± 0.67^a^			
**Negative affect**	
*Pre*	0.32 ± 0.41^b^	0.29 ± 0.50^b^	0.28 ± 0.40	*F*(1,25) = 8.824	*F*(2,50) = 5.430^∗^	*F*(2,50) = 12.335^∗∗^
*Post*	0.18 ± 0.33	0.87 ± 1.01	0.58 ± 0.84			
*Delta*	-0.14 ± 0.21	0.58 ± 0.69^a^	0.29 ± 0.74^a^			
**Fatigue**	
*Pre*	0.86 ± 0.69^b^	0.76 ± 0.63^b^	0.69 ± 0.65	*F*(1,25) = 2.345	*F*(2,50) = 2.117	*F*(2,50) = 12.106^∗∗^
*Post*	0.55 ± 0.55	1.15 ± 0.87	1.00 ± 0.81			
*Delta*	-0.31 ± 0.56	0.40 ± 0.63^a^	0.31 ± 0.71^a^			


*^∗^*p* < 0.05; ^∗∗^*p* < 0.001. ^a^Significant *post hoc* comparison with the outdoor walk, applying a Bonferroni’s adjustment of alpha. ^b^Significant *post hoc* pre-post comparison, applying a Bonferroni’s adjustment of alpha. ^c^Significant *post hoc* comparison between the two IVE conditions, applying a Bonferroni’s adjustment of alpha.*

As shown in **Table 3**, ‘cyber sickness’ was consistently associated with negative affective responses: ‘cyber sickness’ was negatively correlated with enjoyment, positive affect, and tranquility, whereas it was positively correlated with negative affect and fatigue (the latter only in the treadmill condition). Significant correlations were found also between different psychological variables and ‘being there,’ ‘realism,’ ‘sense of reality,’ and ‘fascination,’ though with different patterns of association for the sitting and the treadmill condition (**Table 3**).

The different emotional responses experienced in the outdoor walk and the IVE conditions were also found in the qualitative data. For example, participants expressed positive emotions such as feeling “relaxed” and “happy” (*n* = 13) during the outdoor condition, whereas negative emotions such as feeling “stressed” and “tired” were expressed (*n* = 10) in relation to the IVE conditions. Furthermore, the IVE conditions were viewed as “boring” (*n* = 4), compared to “fun” (*n* = 1) and “great/amazing” (*n* = 2) for their experience outdoors. Furthermore, some participants made reference to the physical reactions experienced during the IVE conditions, especially cyber sickness, which was viewed as having had a strong influence on their affective experience (e.g., “*How I felt during the IVE condition – sick and dizzy – [determined my level of enjoyment]*”). Some participants reported, however, that the novelty of trying the IVE technology by itself provided some degree of enjoyment (*n* = 3; e.g., “*Just the fact that you are using virtual reality [made it enjoyable]*”). Only two participants reported that the element of movement in the treadmill conditions elicited more positive affective responses (e.g., “*The sitting IVE was boring … Moving while the video was playing [made it more enjoyable]*”).

## Discussion

Our findings support, in part, the findings of previous studies showing that green exercise experiences in real natural environments, even in brief bouts (i.e., a 10-min walk), can lead to enhanced psychological states (Barton and Pretty, 2010; Bowler et al., 2010; Thompson Coon et al., 2011). We found in fact that the walk in real nature was associated with an enhanced profile of the participants’ emotional state, specifically in relation to a reduction of fatigue and negative affect, alongside high ratings of enjoyment. On the other hand, despite the participants reporting levels of perceived environmental restorativeness (‘fascination’ and ‘being away’) and physical engagement equivalent to those experienced in the real nature walk, alongside reasonably high levels of some aspects of presence (e.g., ‘being there’ and ‘sense of reality’), unlike the walk in real nature the IVEs led to *negative* affective responses. These latter findings differ from those of previous studies that have used *non-immersive* virtual nature in combination with physical activity, i.e., walking on a treadmill or cycling on a stationary bike whilst watching images or videos of nature projected on a screen (Pretty et al., 2005; Plante et al., 2006; Akers et al., 2012; White et al., 2015; Yeh et al., 2017). These studies found in fact that virtual nature can provide psychophysiological benefits such as improvement of affect states and restoration of mental fatigue. However, such benefits are not as large as those that can be obtained in real natural environments, as shown in studies that had participants visiting a real natural environment and/or viewing a video of the same nature (Plante et al., 2006; Mayer et al., 2009; Olafsdottir et al., 2017). Our findings also differ from those found by Valtchanov et al. (2010), which showed restorative effects in subjects who were exposed to an IVE using a HDM. It is, however, important to note some fundamental differences between our study and that of Valtchanov et al. (2010), which are likely to have played a role in the different outcomes of the two studies, especially resulting in our participants being more exposed to risk of incurring in cyber sickness: first, in the Valtchanov et al. (2010) study the participants sat at a computer station and controlled their movements using a mouse, whereas our participants were ‘passive’ observers of a first-person video; secondly, in the Valtchanov et al. (2010) study the HDM used allowed only a 65° vision, therefore not engaging the participants’ *peripheral* vision.

The negative affective responses that emerged in our study seem to be mainly associated with participants’ experience with IVE being commonly disrupted by the occurrence of cyber sickness. Cyber sickness is known to be a common problem with current IVE technology (Nichols et al., 2000), and a number of theories have been proposed to explain why it occurs. In spite of this, to date little is known about how to prevent it. Two of the most well-known theories on cyber sickness are the *sensory conflict theory*, which suggests cyber sickness is mainly caused by conflicting signals received by the visual and vestibular systems, and the *postural instability theory*, which states that long periods without postural control will cause cyber sickness (LaViola, 2000). In the present study, some participants reported that they struggled to maintain postural control during the treadmill condition, suggesting that postural control might indeed have contributed to the development of cyber sickness in some participants. However, triangulation of the qualitative and quantitative data revealed that those participants who reported challenges in maintaining postural control on the treadmill did not consistently report higher ratings of cyber sickness in the treadmill condition, and in all but one case, the ratings were *lower* than in the sitting condition. On the other hand, during the study it was noted that the participants who reported the highest levels of cyber sickness developed it very quickly after starting the IVE sessions. Again, triangulating the quantitative and qualitative data also revealed that, consistent with the sensory conflict theory, complaints of movement lag and flatness (i.e., poor quality of the imaging, including blurriness and lack of depth) were commonly associated with higher ratings of cyber sickness. This might also explain why the element of physical movement (treadmill condition) was unable to attenuate cyber sickness: in this condition, the participants still struggled with movement lag and flatness, which might have triggered a conflict between visual input and the vestibular system. It should be noted, however, that it is likely that inter-individual differences exist in why and how a person develops cyber sickness, and therefore different theories may be applicable to different individuals under diverse conditions (LaViola, 2000).

Our findings show that movement lag and, in particular, cyber sickness, also emerged as factors influencing the participants’ affective responses, the latter being an important component underpinning green exercise behaviors as well as possibly mediating various health outcomes (Calogiuri and Chroni, 2014). Thus, this issue has important implications for studying the effectiveness of IVE technology in green exercise research. In a recent study, Kokkinara et al. (2016) demonstrated that watching a first-person IVE video of someone walking can create an illusory sense of agency (i.e., the subjective awareness of initiating, executing, and controlling an action), inducing a person to perceive that the movement is initiated by him or herself. It seems, however, that the discrepancy between a person’s movements (or lack of movement, as in our sitting condition) and movements observed in the video can nevertheless result in uncomfortable, or even “frustrating” (as some participants defined it), conflicts between the ‘real self’ and the ‘virtual self.’ Cyber sickness had an even more dramatic impact on participants’ psychophysiological responses, and was consistently associated with less enjoyment, reduced tranquility and positive affect, increased fatigue and negative affect, and (in the treadmill condition) higher HR and perceived exertion. The latter was especially surprising. Previous research shows that individuals tend to report higher RPE when walking/running on a treadmill as compared with walking/running outdoors (Harte and Eifert, 1995; Focht, 2009; Calogiuri et al., 2015). In the present study, it was hypothesized that being exposed to the IVE video whilst walking on the treadmill would have mitigated this effect by causing an ‘attentional shift’ from internal feelings of effort toward the virtual environment, which previous research suggests to be the reason for reporting lower RPE when engaging in green exercise as compared with indoor exercise (Harte and Eifert, 1995). The results, however, did not support this expectation. The higher perceived exertion might be linked to cyber sickness, but also the increased feelings of fatigue or the poor postural control that some participants experienced. The latter factor might, especially, have caused the participants to retain the attention focus toward internal feelings (e.g., keeping the balance of controlling the movements), therefore hindering the shift of focus towards the environment. More research is, however, needed in this field to better understand the reasons that underlie such phenomenon.

Despite the impact of cyber sickness and the different psychophysiological responses observed, our findings suggest some important lines of enquiry for future research and application in this area. In particular, we found that the IVE-related ratings of perceived environmental restorativeness (i.e., the extent to which the participants perceived the virtual environment as fascinating and providing the opportunity to experience ‘being away’) were quite consistently associated with the rating of enjoyment the participants assigned to the IVE experiences. Perceived environmental restorativeness has been found to correlate with ratings of enjoyment during green exercise in real natural environments (Calogiuri et al., 2015). Thus, this finding suggests that in future studies it could be possible to elicit greater enjoyment by producing IVE videos showing natural environments with higher restorative value, as compared with the environment used in this particular study. Furthermore, it is likely that, in the relatively near future, technological developments will allow access to HMDs with higher resolution, which might also limit the occurrence of cyber sickness, and its consequent impact on affective responses.

### Strengths and Limitations of the Study

The strength of our study is primarily ascribed to its novelty: to the best of our knowledge, this study is one of few using a HMD in combination with physical activity (i.e., walking on a treadmill), and the very first using such technology to simulate green exercise experiences. The within-subjects experimental design, with two different IVE conditions administered in counter-balanced order preceded by exposure to a corresponding real environment, also represents a strength of our study. Our design might, however, have led to some confounding effects: first, due to the large number of comparisons, we had to apply a restrictive significance level (i.e., Bonferroni’s adjustment), which is likely to have increased the probability of incurring type-II errors; second, varying weather conditions might have influenced the participants’ experience of the outdoor condition and, relatedly, the psychological outcomes. Most importantly, because the technology used in this study is quite novel, specific equipment that would have helped produce a more stable video was not available. We had to adapt a generic handheld stabilizer, but this was not optimal for a 360° camera, which is very light and symmetrical in shape: additional weights had to be added to the stabilizer, and we had to find solutions to avoid it rotating on its own axis. Furthermore, the program used to improve the stabilization of the video in post-production was at an early stage of development. The development of second-generation technology that will better address these challenges will increase possibilities in this field and might produce different findings.

## Conclusion

Using commercially available IVE technology, we were unable to reproduce psychophysiological responses similar to those experienced during green exercise in a real natural environment. The main factors hindering positive psychophysiological responses during IVE-based green exercise were the occurrence of cyber sickness, the poor image quality, and the lack of a holistic engagement with the natural environment. The additional element of physical movement (i.e., walking on a treadmill) provided only limited benefit compared with the sedentary exposure to the virtual nature walk. IVE technology might in future be a useful instrument in green exercise research and promotion, but only if image quality and cyber sickness can be addressed. IVEs reproducing environments with higher restorative value might also contribute to more positive affective responses during IVE-based green exercise.

## Author Contributions

GC was the primary person responsible for the conception of the study and drafted the manuscript. SL and TR participated in the conception of the study, and provided major contributions in the conception of the experimental protocol and creation of the IVEs, respectively. KF and EB provided relevant administrative support, including carrying out the literature review, recruiting and maintaining contacts with the participants, and conducting the data analyses. GC, SL, KF, and EB also carried out the experimentations and data collection. MT provided substantial contributions to revision of the intellectual content and development of the qualitative analysis. All authors contributed to the writing up of the manuscript and have given approval to its final version.

## Conflict of Interest Statement

The authors declare that the research was conducted in the absence of any commercial or financial relationships that could be construed as a potential conflict of interest.

## References

[B1] Akers A. Barton J. Cossey R. Gainsford P. Griffin M. Micklewright D. (2012). Visual color perception in green exercise: positive effects on mood and perceived exertion. *Environ. Sci. Technol.* 46 8661–8666. 10.1021/es301685g 22857379

[B2] Aspinall P. Mavros P. Coyne R. Roe J. (2015). The urban brain: analysing outdoor physical activity with mobile EEG. *Br. J. Sports Med.* 49 272–276. 10.1136/bjsports-2012-091877 23467965

[B3] Barton J. Pretty J. (2010). What is the best dose of nature and green exercise for improving mental health? A multi-study analysis. *Environ. Sci. Technol.* 44 3947–3955. 10.1021/es903183r 20337470

[B4] Bodin M. Hartig T. (2003). Does the outdoor environment matter for psychological restoration gained through running? *Psychol. Sport Exerc.* 4 141–153. 10.1016/s1469-0292(01)00038-3

[B5] Borg G. A. (1982). Psychophysical bases of perceived exertion. *Med. Sci. Sports Exerc.* 14 377–381. 10.1249/00005768-198205000-00012 7154893

[B6] Bowler D. E. Buyung-Ali L. M. Knight T. M. Pullin A. S. (2010). A systematic review of evidence for the added benefits to health of exposure to natural environments. *BMC Public Health* 10:456. 10.1186/1471-2458-10-456 20684754 PMC2924288

[B7] Calogiuri G. Chroni S. (2014). The impact of the natural environment on the promotion of active living: an integrative systematic review. *BMC Public Health* 14:873. 10.1186/1471-2458-14-873 25150711 PMC4246567

[B8] Calogiuri G. Evensen K. Weydahl A. Andersson K. Patil G. Ihlebaek C. (2016a). Green exercise as a workplace intervention to reduce job stress. Results from a pilot study. *Work* 53 99–111. 10.3233/wor-152219 26684708

[B9] Calogiuri G. Nordtug H. Weydahl A. (2015). The potential of using exercise in nature as an intervention to enhance exercise behavior: results from a pilot study. *Percept. Mot. Skills* 121 350–370. 10.2466/06.PMS.121c17x0 26348226

[B10] Calogiuri G. Patil G. G. Aamodt G. (2016b). Is green exercise for all? A descriptive study of green exercise habits and promoting factors in adult Norwegians. *Int. J. Environ. Res. Public Health* 13:E1165. 10.3390/ijerph13111165 27886098 PMC5129375

[B11] Ekkekakis P. Parfitt G. Petruzzello S. J. (2011). The pleasure and displeasure people feel when they exercise at different intensities: decennial update and progress towards a tripartite rationale for exercise intensity prescription. *Sports Med.* 41 641–671. 10.2165/11590680-000000000-00000 21780850

[B12] Focht B. C. (2009). Brief walks in outdoor and laboratory environments: effects on affective responses, enjoyment, and intentions to walk for exercise. *Res. Q. Exerc. Sport* 80 611–620. 10.1080/02701367.2009.10599600 19791648

[B13] Gale N. K. Heath G. Cameron E. Rashid S. Redwood S. (2013). Using the framework method for the analysis of qualitative data in multi-disciplinary health research. *BMC Med. Res. Methodol.* 13:117. 10.1186/1471-2288-13-117 24047204 PMC3848812

[B14] Gladwell V. F. Brown D. K. Wood C. Sandercock G. R. Barton J. L. (2013). The great outdoors: how a green exercise environment can benefit all. *Extrem Physiol. Med.* 2:3. 10.1186/2046-7648-2-3 23849478 PMC3710158

[B15] Harte J. L. Eifert G. H. (1995). The effects of running, environment, and attentional focus on athletes’ catecholamine and cortisol levels and mood. *Psychophysiology* 32 49–54. 10.1111/j.1469-8986.1995.tb03405.x 7878169

[B16] Hartig T. Evans G. W. Jamner L. D. Davis D. S. Garling T. (2003). Tracking restoration in natural and urban field settings. *J. Environ. Psychol.* 23 109–123. 10.1016/s0272-4944(02)00109-3 27258294

[B17] Hartig T. Korpela K. Evans G. W. Garling T. (1997). A measure of restorative quality in environments. *Scand. Hous. Plan. Res.* 14 175–194. 10.1080/02815739708730435

[B18] Hartig T. Mang M. Evans G. W. (1991). Restorative effects of natural environment experiences. *Environ. Behav.* 23 3–26. 10.1177/0013916591231001

[B19] Hug S. M. Hartig T. Hansmann R. Seeland K. Hornung R. (2009). Restorative qualities of indoor and outdoor exercise settings as predictors of exercise frequency. *Health Place* 15 971–980. 10.1016/j.healthplace.2009.03.002 19427807

[B20] Jaffe D. L. Brown D. A. Pierson-Carey C. D. Buckley E. L. Lew H. L. (2004). Stepping over obstacles to improve walking in individuals with poststroke hemiplegia. *J. Rehabil. Res. Dev.* 41 283–292. 10.1682/JRRD.2004.03.0283 15543446

[B21] Kaplan R. (1989). *The Experience of Nature: A Psychological Perspective.* Cambridge: Cambridge University Press.

[B22] Kaplan S. (1995). The restorative benefits of nature - Toward an integrative framework. *J. Environ. Psychol.* 15 169–182. 10.1016/0272-4944(95)90001-2

[B23] Kokkinara E. Kilteni K. Blom K. J. Slater M. (2016). First person perspective of seated participants over a walking virtual body leads to illusory agency over the walking. *Sci. Rep.* 6:28879. 10.1038/srep28879 27364767 PMC4929480

[B24] LaViola J. J. Jr. (2000). A discussion of cybersickness in virtual environments. *Newsl. ACM SIGCHI Bull.* 32 47–56. 10.1145/333329.333344

[B25] Lee A. C. Maheswaran R. (2011). The health benefits of urban green spaces: a review of the evidence. *J. Public Health* 33 212–222. 10.1093/pubmed/fdq068 20833671

[B26] Litleskare S. Fagerheim K. Holthe G. R. Rydgren T. L. Brambilla E. Calogiuri G. (2017). “Nature experiences in Immersive Virtual Environments (IVE): a new concept for green-exercise and health promotion research,” in *Proceedings of the Annual Conference of the European College of Sport Sciences, 4th-7th July 2017*, Essen 10.13140/RG.2.2.33625.83042

[B27] Loomis J. M. Blascovich J. J. Beall A. C. (1999). Immersive virtual environment technology as a basic research tool in psychology. *Behav. Res. Methods Instrum. Comput.* 31 557–564. 10.3758/BF03200735 10633974

[B28] Lox C. L. Jackson S. Tuholski S. W. Wasley D. Treasure D. C. (2000). Revisiting the measurement of exercise-induced feeling states: the physical activity affect scale (PAAS). *Meas. Phys. Educ. Exerc. Sci.* 4 79–95. 10.1207/S15327841Mpee0402_4

[B29] Mayer F. S. Frants C. M. Bruehlman-Senecal E. Dolliver K. (2009). Why is nature beneficial?: the role of connectedness to nature. *Environ. Behav.* 41 607–643. 10.1177/0013916508319745

[B30] Nichols S. Haldane C. Wilson J. R. (2000). Measurement of presence and its consequences in virtual environments. *Int. J. Hum. Comput. Stud.* 52 471–491. 10.1006/ijhc.1999.0343 11540397

[B31] Olafsdottir G. Cloke P. Vögele C. (2017). Place, green exercise and stress: an exploration of lived experience and restorative effects. *Health Place* 46(Suppl. C), 358–365. 10.1016/j.healthplace.2017.02.006 28270319

[B32] Pasco D. (2013). The potential of using virtual reality technology in physical activity settings. *Quest* 65 429–441. 10.1080/00336297.2013.795906

[B33] Peruzzi A. Cereatti A. Della Croce U. Mirelman A. (2016). Effects of a virtual reality and treadmill training on gait of subjects with multiple sclerosis: a pilot study. *Mult. Scler. Relat. Disord.* 5 91–96. 10.1016/j.msard.2015.11.002 26856951

[B34] Plante T. G. Cage C. Clements S. Stover A. (2006). Psychological benefits of exercise paired with virtual reality: outdoor exercise energizes whereas indoor virtual exercise relaxes. *Int. J. Stress Manag* 13 108–117. 10.1037/1072-5245.13.1.108

[B35] Pretty J. Griffin M. Sellens M. Pretty C. (2003). *Green Exercise: Complementary Roles of Nature, Exercise and Diet in Physical and Emotional Well-Being and Implications for Public Health Policy.* Colchester: University of Essex.

[B36] Pretty J. Peacock J. Sellens M. Griffin M. (2005). The mental and physical health outcomes of green exercise. *Int. J. Environ. Health Res.* 15 319–337. 10.1080/09603120500155963 16416750

[B37] Rogerson M. Gladwell V. F. Gallagher D. J. Barton J. L. (2016). Influences of green outdoors versus indoors environmental settings on psychological and social outcomes of controlled exercise. *Int. J. Environ. Res. Public Health* 13:363. 10.3390/ijerph13040363 27023580 PMC4847025

[B38] Russell J. A. (1980). A circumplex model of affect. *J. Pers. Soc. Psychol.* 39 1161–1178. 10.1037/h0077714

[B39] Sheik-Nainar M. A. Kaber D. B. (2007). The utility of a virtual reality locomotion interface for studying gait behavior. *Hum. Factors* 49 696–709. 10.1518/001872007X215773 17702221

[B40] Slater M. Usoh M. Steed A. (1995). Taking steps: the influence of a walking technique on presence in virtual reality. *ACM Trans. Comput. Hum. Interact.* 2 201–219. 10.1145/210079.210084

[B41] Slater M. Wilbur S. (1997). A framework for immersive virtual environments (FIVE): speculations on the role of presence in virtual environments. *Presence* 6 603–616. 10.1162/pres.1997.6.6.603

[B42] Smith J. W. (2015). Immersive virtual environment technology to supplement environmental perception, preference and behavior research: a review with applications. *Int. J. Environ. Res. Public Health* 12 11486–11505. 10.3390/ijerph120911486 26378565 PMC4586687

[B43] Thompson Coon J. Boddy K. Stein K. Whear R. Barton J. Depledge M. H. (2011). Does participating in physical activity in outdoor natural environments have a greater effect on physical and mental wellbeing than physical activity indoors? A systematic review. *Environ. Sci. Technol.* 45 1761–1772. 10.1021/es102947t 21291246

[B44] Valtchanov D. Barton K. R. Ellard C. (2010). Restorative effects of virtual nature settings. *Cyberpsychol. Behav. Soc. Netw.* 13 503–512. 10.1089/cyber.2009.0308 20950174

[B45] White M. Elliott L. Taylor T. Wheeler B. Spencer A. Bone A. (2016). Recreational physical activity in natural environments and implications for health: a population based cross-sectional study in England. *Prev. Med.* 91 383–388. 10.1016/j.ypmed.2016.08.023 27658650

[B46] White M. P. Pahl S. Ashbullby K. J. Burton F. Depledge M. H. (2015). The effects of exercising in different natural environments on psycho-physiological outcomes in post-menopausal women: a simulation study. *Int. J. Environ. Res. Public Health* 12 11929–11953. 10.3390/ijerph120911929 26404351 PMC4586716

[B47] WHO (2017). *Physical Activity – Fact Sheet.* Available at: http://www.who.int/mediacentre/factsheets/fs385/en/ [accessed February, 2017].

[B48] Yeh H.-P. Stone J. A. Churchill S. M. Brymer E. Davids K. (2017). Physical and emotional benefits of different exercise environments designed for treadmill running. *Int. J. Environ. Res. Public Health* 14:752. 10.3390/ijerph14070752 28696384 PMC5551190

